# External rotation during elevation of the arm

**DOI:** 10.3109/17453670903171867

**Published:** 2009-08-01

**Authors:** Hiroaki Inui, Takashi Hashimoto, Katsuya Nobuhara

**Affiliations:** Nobuhara Hospital and Institute of BiomechanicsTatsunoshi, HyogoJapan

## Abstract

**Background** Knowledge about the pattern of rotation during arm elevation is necessary for a full understanding of shoulder function, and it is also useful for planning of rehabilitation protocols to restore range of motion in shoulders in disorder. However, there are insufficient in vivo data available.

**Methods** We investigated dynamic arm rotation during elevation in different planes using 30 shoulders in 15 healthy men (age range 21–33 years). Both arms were moved from neutral dependent position to maximum elevated position in 4 planes from laterally to anteriorly, and each dynamic course of motion was traced using a 3-dimensional motion capture system.

**Results** Patterns of rotation were categorized as being one of 2 types, depending on whether or not external rotation peaked before the arm reached the maximum elevated position. External rotation peaked at 122˚ (SD14) of abduction, then decreased according to the arm movement in the lateral planes, but increased gradually to maximum elevated position in the anterior planes. Mean maximal angles of external rotation (in degrees) during elevation were 27 (SD11), 13 (SD13), 3 (SD9), and 3 (SD5), from laterally to anteriorly.

**Interpretation** There were differences in rotational patterns, and more external rotation was needed to reach maximum elevation in lateral planes than in anterior planes.

## Introduction

Range of arm motion widens 3-dimensionally and position is determined by angles of abduction, horizontal abduction, and axial rotation. These angles are considered to relate to each other, as suggested by some cadaveric experiments (Browne et al. 1990, An et al. 1991, Moskal et al. 1999). In addition, shoulder disorders can cause excessive or limited external rotation, affecting joint motion and stability (Fleisig et al. 1996, Kuhn et al. 2005, Levine et al. 2007, Limpisvasti et al. 2008). Thus, many in vitro and in vivo studies have investigated relationships between external rotation and position (Stokdijk et al. 2003, Huffman et al. 2006, Mologne et al. 2008, Shafer et al. 2008). However, measurement of maximal external rotation in only 1–2 positions is common practice and the dynamic course of arm rotation during elevation in different planes has not been clarified. Information about rotational pattern could be practical for evaluation of shoulder function, and could prove useful for planning of rehabilitation protocols to restore range of motion for shoulders in disorder or after surgery. We traced dynamic arm movement during elevation in different planes using a 3-dimensional (3D) motion-capture system.

## Methods

**Figure 1:**
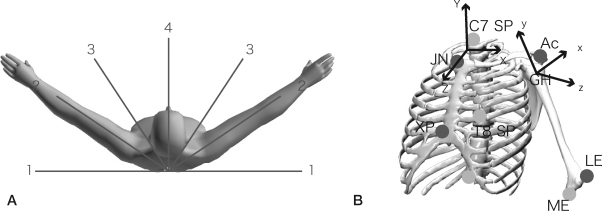
A. Both arms were moved symmetrically from the dependent position with neutral rotation to the maximum elevated position in 4 different planes, referring to tapes attached to the floor at regular intervals of 30°. B. Local thoracic and humeral coordinate systems were defined using bony landmarks. The local thoracic system (X, Y, Z) is defined as: Y = ((JN + C7) / 2 – (XP + T8) / 2) / ∣((JN + C7) / 2 – (XP + T8) / 2)∣; X: perpendicular to plane JN, C7, (XP + T8) / 2; and Z: perpendicular to Y and X. The local humeral coordinate system (x, y, z) is defined as: y = (GH – (ME + LE) / 2) / ∣(GH – (ME + LE) / 2)∣; z: perpendicular to y and LE – ME; and x: perpendicular to y and z. Abbreviations are as defined in the text.

30 shoulders in 15 healthy men were examined. Mean age of the subjects was 31 (21–33) years. The Qualisys ProReflex System (Qualisys Medical AB, Gothenburg, Sweden) including 7 charge-coupled device cameras was used to analyze reflecting markers attached to bilateral epicondyles of the elbow, the lateral side of the acromion on both arms, and 4 sites of the trunk—including the C7 and T8 spinous processes, the xiphoid process, and the jugular notch of the sternum. Passive ranges were evaluated in each subject with a goniometer (Table 1). Both arms were moved symmetrically from the dependent position with neutral rotation to the maximum elevated position and vice versa, in 4 planes. Subjects were instructed to abduct the arms by referring to tapes attached to the floor, at regular intervals of 30˚ (0˚, 30˚, 60˚, and 90˚ anterior to coronal plane) to standardize the amount of horizontal abduction (Figure 1A). Each path was given a number from 1 to 4, starting with the coronal plane. The amount of pronation or supination of the forearm was not specified. One cycle of abduction required an average of 2.3 (2.1–2.4) seconds. Those motions were captured at 500 frames per second and 3D images were displayed and investigated on a computer screen using Qualisys Track Manager tracking software. Before each participant conducted a series of movements, calibration was performed to confirm that error remained within 2 mm.

**Table 1.** Passive range of motion (degrees) for the left and right shoulders in 15 men. Values are mean (SD).

	Flexion	Abduction	Extension at 0° of abduction)	External rotation	External rotation (at 90° of abduction)
R	156 (9)	144 (16)	73 (11)	73 (12)	112 (9)
L	157 (7)	147 (16)	74 (12)	68 (19)	109 (11)

The glenohumeral center was defined as the point located cranially from the marker on the acromion at a distance of 15% of the humeral length, as described elsewhere (Ebara et al. 1998, Nakamura et al. 2004). Local thoracic and humeral coordinate systems were defined with reference to the study by Meskers et al. (1998), as follows (Figure 1B). The local thoracic system (X, Y, Z): Y = ((JN + C7) / 2 – (XP + T8) / 2) / ∣((JN + C7) / 2 – (XP + T8) / 2)∣, where X is perpendicular to the plane JN, C7, (XP + T8) / 2, Z is perpendicular to Y and X, XP and JN represent the xiphoid process and jugular notch of the sternum, respectively, and C7 and T8 are the spinous processes of the seventh cervical and eighth thoracic vertebrae, respectively. The local humeral coordinate system (x, y, z): y = (GH – (ME + LE) / 2) / ∣(GH – (ME + LE) / 2)∣, where z is perpendicular to y and LE-ME, and x is perpendicular to y and z. GH is the center of the glenohumeral joint, and ME and LE are the medial and lateral epicondyles of the humerus. Rotation matrices of the humerus were decomposed into Euler angles. To determine whether the arm of each participant was abducted or adducted along different paths, angles of horizontal abduction and abduction were analyzed. To determine contributions of axial rotation to abduction along each path, relationships between angles of axial rotation and abduction were analyzed.

The shoulders of 8 other healthy men (mean age 28 (17–32) years) were studied using an open MRI system (Magnetom Open; Siemens, Germany) to evaluate the validity of data of the glenohumeral center. Shoulders with the arm neutrally rotated at the side and maximally elevated were scanned using a 3D gradient echo sequence (TR, 56 ms; TE, 25 ms; flip angle, 40˚) with a 2-mm section thickness. Images were digitized to a computer (O2; Silicon Graphics, CA) in which 3D images were constructed using 3D-Virtuoso software (Siemens). Head center and axis of the humeral bone were analyzed as previously described (Inui et al. 2002). 2 cross sections of the humerus were obtained at 3 and 6 inches from the proximal end. The centers for these 2 cross sections of cortical bone were determined by fitting a circle, and the humeral axis was defined as the line passing through these centers. Using the data of Ianotti et al. (1992), showing correlations between size of the glenoid and the radius of curvature of the humeral head, each humeral radius was calculated as follows: radius (mm) = 24 × length of glenoidal long axis / 39 (where 24 is the average head radius and 39 is the average glenoidal long axis). The head was cut by the plane perpendicular to the humeral axis at the distance of the radius from the proximal end, and the center was determined by fitting a circle of the same radius, to be regarded as the head center. We investigated the extent to which the estimated glenohumeral joint center and humeral axis differed from the head center and axis of the humeral bone in each image.

### Statistics

Differences between measurements of each participant were evaluated using the Friedman test. When p-values derived with the Friedman test were significant, the Wilcoxon signed-rank test was used to determine which measurements differed statistically from the others. Values of p < 0.05 were considered statistically significant.

## Results

Horizontal abduction angles of all participants at 60˚, 90˚, and 120˚ of abduction differed statistically significantly along 4 paths (Table 2).

**Table 2.** Mean (SD) angles of horizontal abduction at 60°, 90°, and 120° of abduction along 4 paths.

	Abduction angle		
Path no.	60˚	90˚	120˚
1	10 (7)	14 (6)	22 (8)
2	42 (10)	43 (10)	40 (8)
3	54 (9)	59 (9)	45 (6)
4	64 (6)	77 (8)	50 (8)
P-value	< 0.001	< 0.001	< 0.001
(Friedman test)			

The measured values of horizontal abduction were significantly different from each other (Wilcoxon test).

**Figure 2.**
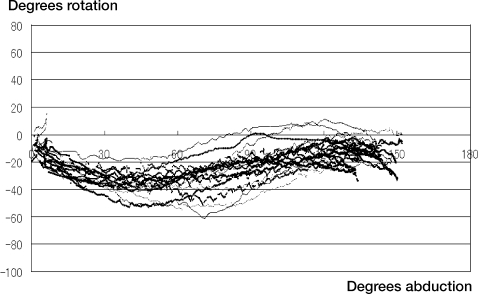

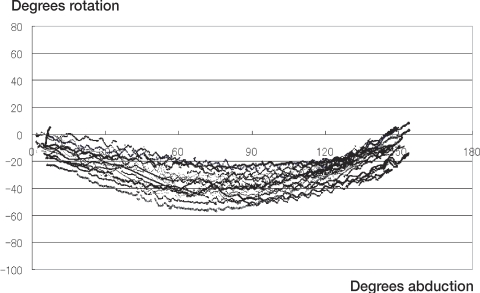
A. Rotational angles of the right arm during abduction in the first plane (coronal plane) representing type A. B. Rotational angles of the right arm during abduction in the fourth plane (sagittal plane) representing type B.

All arms were rotated internally at the beginning, and were then externally rotated. Rotational patterns were divided into 2 types depending on whether external rotation peaked before the arm reached the maximum elevated position. After some degree of internal rotation, type A external rotation started at an average of 53˚ (SD14) of abduction, while type B started at an average of 80˚ (SD13) of abduction. Each wave of type A then peaked at an average of 122˚ (SD14) of abduction and the rotational angle decreased slightly until the arm reached maximum abduction. Waves of type B showed no peak of external rotation. Figure 2 shows rotational patterns during abduction in the first and fourth planes, representing types A and B. Mean rotational angles of types A and B at 60˚, 90˚, and 120˚ of abduction are shown in Table 3. Waves of type A accounted for all participants in the first plane and 8 participants in the second plane, while waves of type B accounted for the other 7 participants in the second plane, and all participants in the third and fourth planes. Both arms of each participant showed the same type in each plane. Maximal angles above 20˚ of abduction during the elevation of each path were compared, avoiding the gimbal-lock problem. These values averaged 27˚ (SD11) in the first plane, 13˚ (SD13) in the second plane, 3˚ (SD9) in the third plane, and 3˚ (SD5) in the fourth plane, showing that the amount of external rotation needed to reach maximum elevation was significantly greater when the arm was elevated along more horizontally abducted paths (Table 4).

**Table 3.** Mean (SD) angles of axial rotation at 60°, 90°, and 120° (both types).

	Abduction angle		
Type	60˚	90˚	120˚
A	–2 (10)	6 (12)	14 (14)
B	–9 (7)	–8 (10)	–3 (11)

**Table 4.** Maximal angles (SD) of external rotation during the elevation along 4 paths.

Path no.	Mean (SD)	p-value **^a^**
1	27 (11)	< 0.001
2	13 (13)	< 0.001
3	3 (9)	0.4
4	3 (5)	

**^a^** Wilcoxon test.

Distance between the estimated center of the glenohumeral joint and the anatomical head center was increased in the maximum elevated position compared to the dependent position. Estimated centers deviated at a mean distance of 3.4 (SD 0.5) cm in the maximum elevated position and 1.3 (SD 0.5) cm in the dependent position. Mean angles between estimated axis and bone axis were 2.6˚ (SD 0.6) and 2.5˚ (SD 0.7), respectively.

## Discussion

Angles of arm abduction, horizontal abduction, and axial rotation are considered to be related to each other, as suggested by various cadaveric experiments (Browne et al. 1990, An et al. 1991, Moskal et al. 1999). However, insufficient in vivo data have been available and the relationships between them have thus remained unclear. We traced dynamic arm movement during elevation in different planes.

The rotational relationship of the glenohumeral joint differs between maximum elevation and arm at side, as noted by Pearl et al. (1993), who showed a 20˚ difference in rotational angle of the arm using a global diagram. In addition, range of axial humeral rotation decreases as maximum elevation is approached. Given this rotational gap and confined range of rotation when the arm is elevated, the humerus would have to rotate externally to elevate from the dependent position.

“Codman's paradox” (Codman 1934, Cheng 2006) indicated that amounts of external rotation would differ between planes of elevation by showing the phenomenon of the humerus rotating naturally. With the arm elevated forward (i.e. in the sagittal plane), the humeral axis is tilted forward on the glenoid and the glenohumeral joint rotates naturally by elevation (Kapandji 1970). Natural external rotation by elevation would compensate for the gap between the dependent and maximum elevated positions and no rotation around the humeral axis might be needed by forward elevation. This may be the reason for the amount of change in the angle of rotation from 20˚ of abduction to almost 0˚ in the third and fourth planes. Conversely, when the arm is elevated in the scapular to coronal planes, the humerus would need to rotate around the axis to compensate for the rotational gap between positions. However, “Codman's paradox” cannot explain different patterns in external rotation, even though this can explain different amounts of change in the angle of rotation during elevation between the planes.

Using a magnetic tracking system, Stokdijk et al. (2003) noted different patterns of glenohumeral rotation due to the plane of elevation. Even though that study involved a static method, they also showed that external rotation peaked and then decreased as the arm reached maximum elevation in the coronal plane. The fact that both their static studies and our active studies showed the same pattern indicates that the peak in external rotation is induced by tension in the capsule, not by active force. When the arm is elevated in the coronal plane, the inferior glenohumeral complex reportedly becomes tight and supports the humeral head like a hammock (O'Brien et al.1990). Thus, it is possible that the rotational pattern in the coronal plane reflects the function of the inferior glenohumeral complex. Even though the pattern of rotation was the same in the coronal plane, angles of abduction at its peak were different between subjects. We thought shoulders with lax inferior glenohumeral complex should be more raised to show the peak in external rotation. However, we only examined range of motion in each subject, and joint laxity was not quantified. Further studies including shoulders with laxity or instability would be of interest.

Our study had some limitations. We analyzed reflecting markers glued to the skin on anatomical landmarks. Palpation of bony landmarks is a non-invasive method to determine joint kinematics without exposure to radiation. The reliability of such data has been evaluated (Van der Helm and Pronk 1995, De Groot^ 1997^). Active rotational movement of the scapula is difficult to detect and glenohumeral motion cannot be traced. Some estimation is also needed for tracing the proximal part of the humerus. Some authors (Ebara et al. 1998, Nakamura et al. 2004) have defined the proximal part of the humerus as the point cranial to the marker on the acromion, which was also estimated as the center of the glenohumeral joint. This estimation could facilitate analysis of humeral motion, but some degree of error is undoubtedly caused by rotation or tilting of the scapula. We also evaluated those errors when analyzing the head center and humeral axis in 3D computer-generated MRI, with the arm in elevated and dependent positions. Errors of angle in the humeral axis appeared to be within 5˚, which is about the same as the palpation error in angles of abduction and horizontal abduction of the humerus in the previous study (De Groot^ 1997^). Scapular motion is crucial for normal shoulder mechanics, and altered scapular orientation was reportedly important in some shoulder disorders (Ludewig et al. 2000, Myers et al. 2005, Meyer et al. 2008). We must also admit that information was lost when motion was not recorded in the glenohumeral and the thoracoscapular joints individually. In the future, we hope that the new methodology will enable us to evaluate the dynamic kinematics of the scapula during arm motion.

Even though these errors or limitations should be considered, our method appears sufficient to allow determination of rotational patterns in relation to elevation plane. Such information might be useful for clinicians planning rehabilitation protocols for shoulders in disorder or after surgery. When angles of both external rotation and abduction are limited, arm abduction can be recovered more effectively using anterior paths than using lateral paths, because anterior paths require less external rotation without peaking before the arm reaches maximum elevation.
